# “*Tie your camel first, then rely on God*”: reconceptualizing Javanese Islamic values to support palliative care at home

**DOI:** 10.1186/s12904-024-01383-w

**Published:** 2024-03-02

**Authors:** Raditya Bagas Wicaksono, Amalia Muhaimin, Dick L. Willems, Jeannette Pols

**Affiliations:** 1 Department of Ethics, Law, and Humanities, Amsterdam University Medical Center, University of Amsterdam, Meibergdreef 9, Amsterdam, The Netherlands; 2 Department of Bioethics and Humanities, Faculty of Medicine, Universitas Jenderal Soedirman, Purwokerto, Indonesia; 3 Amsterdam Public Health Research Institute, Amsterdam University Medical Center, Amsterdam, The Netherlands; 4 Department of Anthropology, Faculty of Social and Behavioral Sciences, University of Amsterdam, Amsterdam, The Netherlands

**Keywords:** Islamic values, Javanese, Ethics, Palliative care, Indonesia, Ethnography

## Abstract

**Background:**

In the last decade, there has been a growing concern to make palliative care more culturally sensitive and contextually appropriate. This concern is also relevant in Indonesia, where the progress of palliative care, particularly in home-based care, has been slow. Like elsewhere in the world, there has been a growing awareness of the importance of shifting from a curative orientation towards a palliative one, especially in cases where further medical treatment is futile. In this paper we argue that the development of palliative care practices would benefit greatly from learning about the values that are important for patients, families, and health professionals. It is important to understand these values to support forms of care that aim to enhance quality of life. To demonstrate this, we analyse the care values people in rural Java evoke in their home palliative care practices.

**Methods:**

We conducted an eight-month ethnographic study involving forty-nine patients, families, and health professionals.

**Results:**

We identified three specific Javanese Islamic values: making an effort (*ikhtiar*), being sincere (*ikhlas*), and being in a state of surrender (*pasrah*). These values influenced the participants’ activities in a palliative care setting. Based on our findings, we suggest three strategies to incorporate these values into palliative care practices and to better facilitate palliative care’s integration into Javanese Muslim communities. The first strategy is to include efforts to reduce suffering and improve the quality of life using the concept of *ikhtiar*. The second strategy is to foster sincerity (*ikhlas*) to help patients and families accept the realities of their condition and provide care for patients at home. The last strategy is to clarify that palliative care is not synonymous with ‘giving up’ but can be seen as an act of pious surrender.

**Conclusions:**

Our study identified three Islamic-Javanese values that can be incorporated to strategies aiming at enhancing palliative care practices, resulting in care focused on improving quality of life rather than futile attempts at a cure.

## Introduction

The definition of palliative care provided by the World Health Organization emphasizes the importance of improving quality of life for patients and their families. A palliative approach becomes more important as a disease progresses, while a curative approach slowly loses importance for chronically ill patients with an incurable disease [1]. These conceptualizations, however, originated and developed mainly in Western countries [2]. Over the past decade, there has been a growing concern to make palliative care more culturally sensitive and contextually appropriate. This is considered one of the fundamental elements in delivering holistic and comprehensive care [3–5].

This concern about fostering locally appropriate palliative care is relevant in Indonesia, where palliative care has been progressing slowly [6], particularly in the field of home palliative care. Despite the existence of national guidelines for palliative care which encourage natural death and the withholding of futile treatment [7], and policy regarding the organization of palliative care [8], the implementation of these guidelines still varies and is limited. Furthermore, current Indonesian health policy primarily focuses on curative care [9], with a substantial risk of futile treatment and suffering for palliative patients.

Health professionals are expected to approach their patients with understanding, so that they can win the trust of patients and deliver better care. To improve care, it is important to know peoples’ local values [10]. In this paper, we argue that to further improve palliative care aimed at quality of life, professionals should link these values to local understandings of what good care practices are for chronic and incurable diseases. When health professionals fail to properly understand local values and practices, misunderstandings can arise, leading to perceptions of poor or inadequate care by patients and their families [11]. There is a scarcity of literature exploring the values that are considered important in palliative care for patients, families, and health professionals, and this is certainly the case for home settings in rural Indonesia. We therefore conducted a study on home palliative care using an inductive, ethnographic approach, because this methodology can better capture the local values of patients, families, and health professionals in Indonesia. Through the analysis, we aim to provide conceptual understanding and insights to further develop home palliative care practices, specifically in Java, which is the contextual focus of this study. We develop a comprehensive understanding of the values which frame how families and professionals understand and practice palliative care in Banyumas, Central Java. We explored two key questions: what values are important to health professionals, families, and patients in home palliative care practices; and how can we distil and describe these local values to help shift the focus of attention from providing curative care to delivering palliative care aimed at improving the quality of life? By understanding values and creating a conceptual space to integrate palliative concepts within them, we demonstrate the challenges associated with developing a palliative care approach. Furthermore, we provide suggestions for developing a framework that integrates local values with a palliative concern for quality of life, thereby supporting the development of home palliative care.

## Methods

### Design

This study is part of a larger study which aims to understand the conceptualizations, practices, and family experiences of home palliative care in rural Indonesia. To address our research question, we adopted an ethnographic approach, as this would enable a deeper understanding of the local meanings of values, norms, perceptions, and behaviors among a specific group of people. Through prolonged research in the community, we were able to observe and experience research participants’ practices in authentic settings. These advantages of ethnography justified its suitability for our study [12, 13]. To ensure the quality of this study, we strive to stay as close as possible to the local words and context, preserving sensitive and crucial meanings that are not easily translatable. The specificities of local terminologies found in our study will help improve understanding of different approaches of care practices [14], and to help readers reflect how these findings are similar or different to their particular setting [15].

### Setting

We conducted an ethnographic study in Banyumas, a region located in the south-western part of Central Java. Banyumas is situated at the foot of Mount Slamet, an area with fertile soil, and the people in this region, particularly in rural areas, rely heavily on agricultural activities. The estimated population of Banyumas is 1.8 million. The majority of the population in the region (91%) live in rural villages (*désa*) [16]. Islam is the predominant religion in Banyumas, with approximately 98% following the religion [17]. People in this area speak a unique dialect of Javanese called *Ngapak Banyumasan*, and cultural norms and values in the area include a strong sense of egalitarianism and direct communication (*blakasuta*) [18].

The health infrastructure in this region is comprised of 4 public hospitals, 19 private hospitals, 40 public primary health center*s* (*Puskesmas*), and 66 primary care clinics [16]. One of the public hospitals serves as a regional referral hospital and has a palliative team, established in 2017. However, knowledge about this team among health professionals in the region is limited. A few home care services are available, provided by private hospitals and clinics and mostly self-funded by the patient or their family. These services predominantly focus on wound care. Banyumas has two medical schools, one of which is a public university. However, neither of these schools includes a specific palliative course or module for medical students during their bachelor's degree or clerkship phase, nor for healthcare practitioners. The exposure to palliative care is currently limited to one-off lectures.

### Participants and recruitment

We used purposive sampling to ensure the representation of patients, families, and healthcare workers from different areas within the region, including rural and suburban areas. We also sought to recruit a diversity of patients in terms of age, disease and gender. Medical doctors with diverse specialties were also involved in this study. These different characteristics of participants were included to ensure different perspectives and reflection of population diversity of the healthcare system were all taken into account [19, 20]. Participants’ recruitments were discontinued after data saturation had been reached, which is a state where new data repeats what was expressed in previous data [21].

The criteria for inclusion and exclusion of participants were applied prior to their recruitment, as described in Table 1.

**Table 1:** Inclusion and exclusion criteria

**Inclusion criteria**	**General criteria:** - Being 18 years of age or older **Specific criteria:** Patient: - Diagnosed with life-threatening or life-limiting disease Receiving home care from a healthcare facility Family: - Being the spouse, child, parent, or relative responsible for the patient’s care - Already providing care for the patient for at least one month - Living with the patient or providing daily care to the patient Healthcare workers: - General practitioner, specialist, nurse, midwife, or other healthcare professionals involved in the care of patients with life-threatening or life-limiting diseases Institutional representative: - Regional office of the national health insurance (BPJS) organization, regional health department, and a health philanthropic organization
**Exclusion criteria**	For in-depth interviews: - Experiencing difficulty in comprehending and communicating clearly For in-depth interviews and observations: - Patients with clinically unstable conditions

The patients were recruited based on the recommendations of healthcare providers, including hospitals and *Puskesmas*. We asked for patients who were in a palliative state and receiving home visits or care by healthcare professionals. After meeting the patients, we confirmed their eligibility for inclusion in the study. Four patients were recommended by the home care unit of a private hospital, one from a local referral hospital, one from a philanthropic organization, and the remaining patients were recommended by several *Puskesmas* in the region. Healthcare workers were recruited from the local referral hospital, private hospitals offering home care services, a philanthropic organization, and several *Puskesmas*. The institutional representatives involved in this study were officially appointed by the institution through formal correspondence. The ethnographer approached each eligible participant with information and a request to participate in the study.

### Data collection

We conducted eight months of fieldwork from June 2022 until January 2023. The first author of this study served as an ethnographer and travelled to various parts of the region, including rural areas of Banyumas, with motorcycle or car for transportation. The distance between the patients' homes and the city center ranged from 2 km to 37,1 km. In-depth interviews with patients and families were conducted at the patients’ homes, with an average duration of 50 min. The ethnographer also observed how care was provided at home. Multiple visits were made to each family unit to capture the care provision at home, whether by healthcare workers (nurses and midwives) or by family caregivers. In the healthcare system of our setting, midwives were involved in palliative care due to their nursing and communication skills, and their closeness with local communities. Therefore, we included midwives in this study.

The duration of visits (interview and observation) ranged from one hour to three hours per day, repeated two to three times as needed. Field notes, photographs, and audio recordings were taken during the visits and interviews. Shortly after each visit, the field notes were converted into detailed descriptions. After initial findings were generated, both a literature study and further conceptual analyses were conducted to explore and clarify the values and concepts discussed by the participants. The literature study involved religious books such as the *Quran* and *Hadith*, relevant journals, and an Indonesian official dictionary that contained the words *ikhlas, ikhtiar,* and *pasrah*.

### Data analysis

Thematic analysis, as described by Braun and Clarke [22], was used in this study. We conducted an inductive analysis by drawing insights directly from the interview transcripts and field notes, but closely informed by the research question. The analysis was conducted iteratively during the data collection process to capture emerging data and adjust the fieldwork guide. Two researchers (RBW and AM), both proficient in both Bahasa Indonesia and Bahasa Jawa, carried out the coding of the data and engaged in discussions to interpret the results. Regular biweekly meetings involving all members of the research team (RBW, AM, DL, JP) were held to discuss findings, adjust the interview and observation guide as needed, and establish consensus on the codes, categories, and themes. Codes with similar meanings were categorized and compared to each other, leading to the identification of overarching themes.

### Validity

The validity of this study was maintained through several measures. Firstly, data triangulation was pursued by using various data collection methods. Through prolonged engagement with the participants using multiple visits and during home observations, the credibility of this study was strengthened. This approach allowed us to verify the consistency of participants' statements with their actions, increasing confidence in the authenticity of the information provided. All raw data, including field notes, audio recordings, and field pictures, were stored within a single project file of qualitative data analysis software (MAXQDA Plus 2022). Any subsequent changes, notes, or reflections were recorded within the project file, ensuring a transparent audit trail. Field notes were written up in detail immediately after each data collection session to provide a thick description of the interview and what was observed in the field.

### Positionality

The ethnographer is a medical doctor who has been working in private hospitals for four years and in primary care clinics for six years. The ethnographer has a 15-year history of living in the region, enabling him to grasp the local language, expressions, and dialect. Additionally, the ethnographer’s personal experience of coping with family loss caused by cancer has provided a profound understanding of what it means to be a family caregiver for a patient with a terminal illness. This combination of medical expertise, cultural familiarity, personal experience, and insider knowledge contributes to the richness and depth of the ethnographer's understanding and analysis in this study.

## Results

Forty-nine participants were involved in in-depth interviews of this study (see Table 2). We included 12 family units for observation with an equal representation of genders among the patients (see Table 3). More female than male caregivers participated in this study. Eight patients were eligible for in-depth interviews, while others were not interviewed due to their inability to communicate effectively due to their illness. The patients had various diagnoses, including neurologic disorders (such as stroke with complications, cerebral palsy, and paraplegia), diabetes with complications, severe joint disorder, and breast cancer. The complications included pressure ulcers and diabetic ulcers. The purposive selection of various patient diagnoses aimed to capture diverse experiences of care. Three patients did not have a family caregiver available for the interview: one patient only lived with his juvenile son, while others were unavailable due to work obligations.

**Table 2:** Interviewed participants’ demographic data

Characteristics	Number
Gender (*n* = 49)	
- Male	24
- Female	25
Background (*n* = 49)	
- Patients	8
- Family caregivers	10
- General practitioners	6
- Specialists	7
- Nurses	10
- Other healthcare workers	5
- Institutional representatives	3

**Table 3:** Observed patient-family units’ characteristics

Characteristics	Number
Patients’ gender (*n* = 12)	
- Male	**6**
- Female	6
Family caregivers’ gender (*n* = 10)	
- Male	2
- Female	8
Family caregivers’ relationship (*n* = 10)	
- Spouse	3
- Daughter	3
- Mother	2
- Son-in-law	1
- Sister	1
Patient’s disease (*n* = 12)	
- Stroke with complications	4
- Diabetes with complications	3
- Severe joint disorder	2
- Cerebral palsy	1
- Paraplegia	1
- Breast cancer	1

Healthcare workers involved in this study consisted of 17 males and 14 females. Among them were six general practitioners and seven medical specialists from various departments, including oncology, neurology, pulmonology, and cardiology. Additionally, ten nurses, three midwives, one psychologist, and one community health volunteer or cadre (*kader*) were recruited. A *kader* is a person chosen by the community who is trained to encourage voluntary participation in health empowerment [23]. Three institutional representatives were also included to provide insights from healthcare management perspectives. The healthcare workers were recruited from different healthcare facilities in rural and suburban areas of the region. Regarding the participants' religion, we did not specifically seek participants with different religious affiliations, resulting in most participants being Muslims (98%), with only one participant (a midwife) being Christian.

Since the fieldwork is part of a larger research project, there is a rich amount of data related to different research questions. However, in this paper, we focused on three values that emerged from our data that were unique and specific to the research setting: *ikhtiar* (making an effort); *ikhlas* (being sincere), and pasrah (being in a state of surrender). These values were considered Javanese-Islamic values due to their etymology being derived from Arabic and adapted to local language, as well as their particular usage among Javanese communities in Banyumas with a Muslim background. These words were specific to Javanese context and not very commonly used in other Muslim communities.

**Fig. 1:**
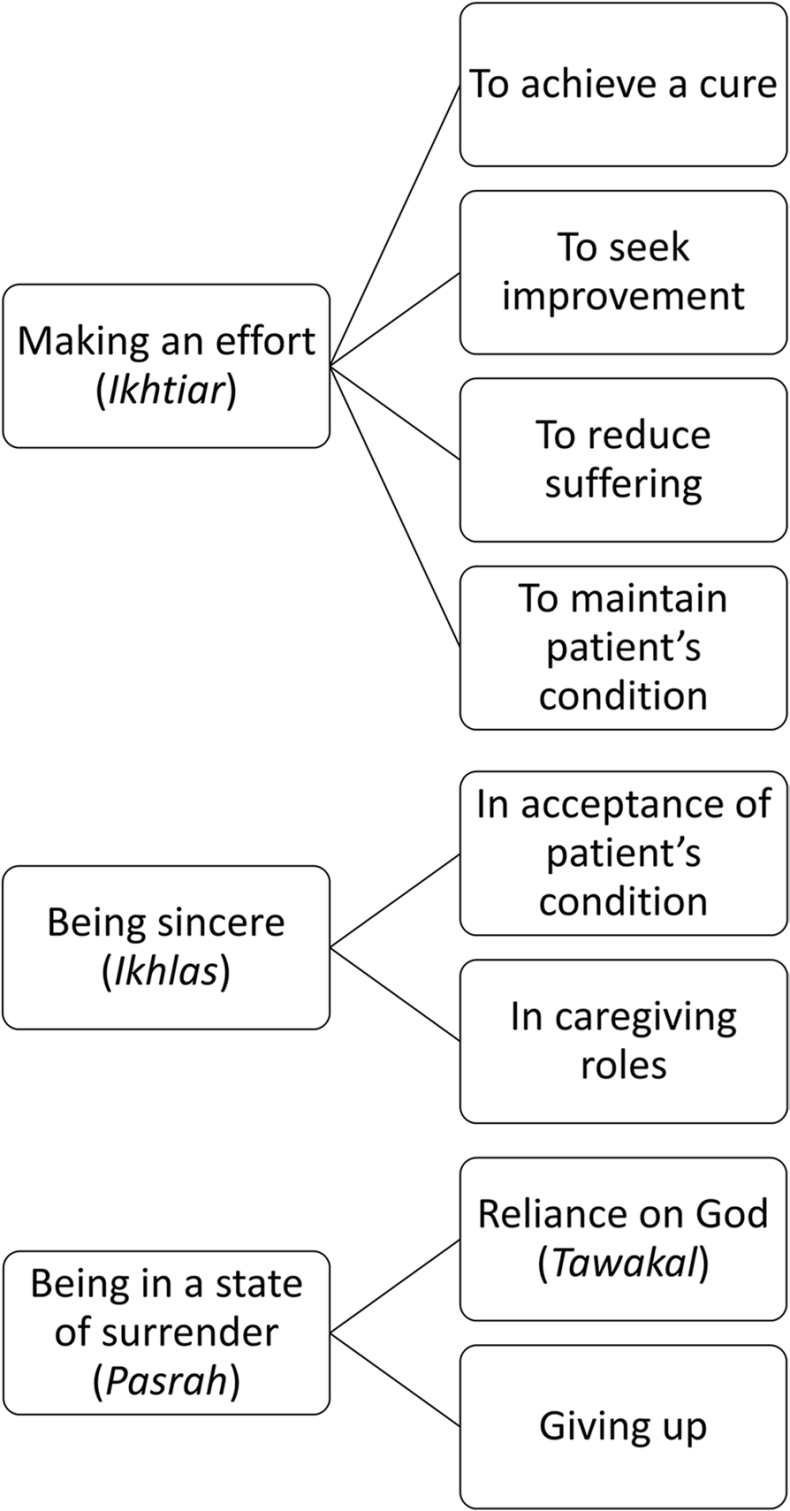
Schematic summary of the themes and categories

Participants demonstrated different goals for *ikhtiar,* including achieving a cure, seeking improvement in the patient’s condition, reducing suffering, and maintaining a stable condition. *Ikhlas*, or being sincere, represented a state of mind characterized by the sincerity of accepting a patient’s condition, as well as willingly taking on caregiving roles. The third theme, *pasrah* or surrender*,* also pertained to a state of mind, which encompasses both ‘reliance on God’ (*tawakkul or ‘tawakal’ in Bahasa Indonesia*) and ‘giving up’. A schematic summary of the results is presented below in Fig. 1.

To better understand these empirical findings from fieldwork, we provide a general explanation of the three values based on the literature study, conducted to interpret the results obtained from the fieldwork.

### General description of Islamic values in Indonesia

*Ikhtiar* is a verb in Bahasa Indonesia adapted from the Arabic word اِخْتِيَار (*iḵhtiyār*). This term is included in the Official Dictionary of Bahasa Indonesia with the meaning of ‘making an effort (to reach a specific goal)’ [24]. Although it is now recognized as an Indonesian word, the term “*ikhtiar”* is more commonly used by Islamic Indonesian communities. The process of *ikhtiar*, making an effort, is accompanied by prayers, praying that God will reward one’s effort with the desired result [25, 26], which in the context of this study would mean a cure.

*Ikhlas* is an adjective in Bahasa Indonesia that is identical to Arabic إِخْلَاصٌ (*ikhlāṣ*), translated as 'having purified one's intention’. In the Official Dictionary of Bahasa Indonesia, *ikhlas* means sincerity (*ketulusan*). Another meaning of *ikhlas* is willingness (*kerelaan*) [24]. *Ikhlas*, as defined in theoretical Islamic teachings, means purifying the intention and is not merely associated with acceptance of a certain condition. *Ikhlas* is doing something without seeking worldly benefits or praise. People who are *ikhlas* may hope for a reward directly from God, doing good deeds that bring them closer to God or even lead to a reward of Jannah (paradise) in the afterlife [27].

*Pasrah* is also an adjective in Bahasa Indonesia that means being completely reliant on something [24]. In Islam, this term refers to *tawakal,* particularly with respect to relying on God’s will. *Tawakal* (Arabic: تَوَكُّل, *tawakkul*) means relying on God's plan, but only after making an effort (*ikhtiar*) to change something. An example of the Islamic teaching on *tawakal* as taught by Prophet Muhammad (Peace Be Upon Him) is: “*Tie your camel first, then put your trust in Allah*” [28]. In this prophetic teaching (*hadith*), the Prophet encouraged his followers to trust in God’s plan, but only after they have done everything possible to handle the situation appropriately. Whatever the result, it is then destined by God [29, 30].

### Making an effort (*ikhtiar*/اِخْتِيَار)

*Ikhtiar* was understood or perceived by our participants as making their best effort. This value is defined as an active attempt to achieve something. *Ikhtiar* leads to four different goals, as described by them. The first goal, reported by patients and their families, was their goal to achieve a cure. They wished to witness a complete recovery of the patient, as if nothing had ever happened. An example of this goal was the hope of seeing the patient walk again, just like she could before her stroke.

*Interviewer: What is your main motivation for providing caregiving?*

*Patient’s daughter: My biggest motivation is to see my mother cured. That’s all. (I want her to be) Cured, so she can walk again. That’s why all of us, her children, make every possible effort (ikhtiar) as advised by the doctor. We follow the doctor’s advice because we are not knowledgeable about medical matters (awam secara medis). We don’t know what is right. So, if the doctor says she has to be hospitalized, we will approve this. (F01: 107-108)*

Another example was a breast cancer patient willing to undergo chemotherapy, despite the associated nausea, in order to achieve a cure for the disease. This patient initially presented with an unstable condition and a severe cancer-related wound. Despite the initially unfavorable prognosis, the patient remained motivated to be cured and followed the health professionals’ recommended treatment.

*Interviewer: You have previously undergone chemotherapy six times. If you were advised to undergo it again and experience nausea, will you still proceed with the chemotherapy?*

*Patient: Yes, I will remain motivated because I want to be healed. I am willing to do whatever is necessary as instructed by the doctor. The most important thing for me is to be cured. (P10: 154-157)*

The next goal was to seek an improvement in the patient's condition. Patients, families, and healthcare workers expected various improvements, such as better blood glucose levels or healing of ulcers. This goal held significant importance for certain participants, influencing their decision on whether to continue hospitalization for medical treatment or not. One family caregiver shared her experience of requesting an early discharge from the hospital due to a lack of observed improvement in her husband, who had been diagnosed with a stroke.

*Wife: I asked, "Doctor, can he go home now? The blood pressure is not as high as before." The doctor said, "Well, okay, he can go home now." I immediately took care of the hospital administrative papers and brought him home. Even after being treated in the hospital, he still couldn’t do anything. So, I will try to treat him at home with whatever I can.*

*Interviewer: What is the reason for wanting to go home?*

*Wife: You know, in the hospital, he can only lie on his bed (ngathang-ngathang). What else can I do? It is far from anywhere. It’s difficult to find curcuma (kunir). In the hospital there is no curcuma, right? They only provide drugs and regular food. I will try to treat him with herbs. (F09: 139-141)*

Other participants also described various efforts to seek improvement, including maximizing medical treatment. This effort was made particularly by healthcare workers, such as escalating the therapy based on medical guidelines or consulting with different specialists.

*Interviewer: I am interested in your earlier statement about ikhtiar. Could it be because, as Muslims, we can’t make judgments regarding life expectancy and prognosis, despite survival rates? To what extent should we, as doctors, continue our efforts (ikhtiar) to treat patients?*

*Pulmonologist: It depends on the case, of course. Let’s take the example of a late-stage chronic obstructive pulmonary disease; this condition is clinically difficult to treat. Even when patients have been prescribed different medications, the response is often poor. We try using steroids, anti-muscarinic drugs, beta-agonists, and everything to the maximum extent possible. Perhaps the pulmonary function is already severely compromised. However, I can’t say that the patient is in a terminal stage. I will tell the patient to continue our efforts (ikhtiar), using the prescribed medication optimally, and ask them to pay attention to factors that can cause shortness of breath. I believe paying attention to room ventilation or other factors may be helpful. (D12: 57-58)*

The quote above came from a medical specialist who explained that she had tried to maximize the treatment according to medical guidelines, but the desired result was not achieved. However, she refrained from delivering a negative prognosis and instead encouraged the patients to try something else. This choice demonstrates the importance she placed on continuing to make efforts and engage in *ikhtiar,* with the hope of improving the patient’s condition. It also highlights that this does not necessarily involve solely curative medical treatments but can also encompass efforts to enhance the patient’s quality of life.

Patients and their families pursued the goal of maximizing treatment in various ways, often exploring and combining different approaches, including complementary and alternative medicines. They might try alternatives such as traditional massage or new herbal potions, based on recommendations from friends or information found on the internet.

*Interviewer: Where did you come across the "bunga telang" herbal potion?*

*Patient’s sister: From our physiotherapist, he mentioned that he watched videos about it on YouTube. Whether it is effective or not, I decided to give it a try anyway. (I want to try) Everything. I am experimenting (coba-coba)… Finally, my brother tried drinking it, but not regularly, sometimes in the morning, afternoon, or before bed. Alhamdulillah (praise be to God), his blood glucose has stabilized at around 100 (mg/dl).*

*Interviewer: So, you tried…*

*Patient’s sister: We tried every recommendation, and surprisingly, they worked. But we also continue using the medications prescribed by the doctor... (FP11: 209-214, 226-228)*

The ethnographer’s personal experience during the fieldwork exemplified the complexities of accessing healthcare, as encountered by both health professionals and patients/family members residing in remote parts of the region. Limited healthcare access became one of the barriers they faced in pursuing *ikhtiar,* such as seeking treatment from a recommended traditional massage healer. Travelling to different parts of the region, particularly to remote areas where participants lived, posed challenges, as described in the following field note.

The journey was difficult due to the poor quality of the road. It took me 75 min (average speed of 30 km/hour) to reach the furthest patient. Unclear addresses and signage made me stop and ask locals for directions several times. The topography of the location added another layer of difficulty, with steep hills and multiple U-turns in the foothills of the mountain. Unpredictable weather with heavy tropical rain made it more challenging for me to reach the location, especially when I was riding my motorcycle. (Field note 3).

A third goal for *ikhtiar* described by the participants in this study was reducing patient’s suffering, including pain and symptom control. This goal was described by healthcare workers, particularly those who are more familiar with or trained in palliative care. Healthcare workers could reduce suffering by allowing patients to eat anything they wish and discharging them from the hospital earlier, as they had wished. This option was mainly chosen for patients with a known poor prognosis. Another example of how this goal was pursued was through palliative surgery.

*Interviewer: What do you mean by the patient can experience the disease better (menjalaninya lebih enak)?*

*Surgical oncologist: For example, if the patient is not in pain, she can perform her activities without being limited by her tumor. She can engage normally in daily activities, including drinking and eating, if we can control the condition with palliative care. She can enjoy life better, even though there is a tumor inside her body. Let’s imagine a breast cancer patient with a wound and bone metastasis; she is incurable, right? If there is a skin invasion and the wound becomes persistent, it can become rotten-smelly. If she is invited to a party (kenduren), her friends would be disturbed, "What is this smell?!" One of the palliative care approaches is to control the wound through an operation, removing the tumor and closing the skin, so she can continue her activities and socialize without being limited by the wound. (D10: 57-58)*

Although the doctor stated that the surgery was intended to be palliative, it is possible that patients or families perceived it to be curative. It may have been challenging for the families to differentiate whether the surgery was aimed at curing the underlying disease or only controlling the secondary wound.

Another participant, an emergency room doctor in a referral hospital, shared his experience of not performing aggressive treatments, such as cardiopulmonary resuscitation (CPR) and intubation, even though this decision was not always accepted by the patient’s relatives (extended family). From the following quote, we can understand that while the family sought a cure, the doctor wanted to engage in palliative care and alleviate patient suffering.

*Emergency room doctor: Most Indonesians don’t understand the goals of palliative care, such as providing a humane end-of-life experience for the patient. When I encounter people who are unaware of this goal, sometimes the patient's family can’t accept it when I try to discuss it.*

*Interviewer: Was it like your heart can't handle it?*

*Emergency room doctor: No, personally, I could do it. I would explain to them that the patient is already in the end stage. And there is nothing more we can do. I would recommend to them that instead of performing CPR or intubation, I prefer to focus on reducing the patient’s suffering. That’s the concept of palliative care, right? However, Indonesians generally do not understand that. As a result, palliative care in this hospital can’t be implemented effectively. (D02: 26-28)*

The last goal of *ikhtiar* is to maintain the patient’s condition as stable as possible. To achieve this goal, some patients and their families emphasized the importance of continuing to see the doctor and taking the prescribed medication from the hospital or *Puskesmas*. Others engaged in home physical exercises, such as cleaning the front yard of their own home and walking around the neighborhood. These efforts are made to maintain the stability of the condition and prevent further deterioration.

*Interviewer: How do you feel now that you have kidney failure and need to undergo hemodialysis?*

*Patient: Well, now I just have to do it; as long as I continue with hemodialysis twice a week, follow the dietary restrictions, and avoid consuming too much water and fruit, I believe my condition will remain stable.*

*Interviewer: So, it can be maintained, right?*

*Patient: Yes, I can still carry out my daily activities. I call this “berdikari” (being independent), trying to take care of myself. If there are complications, it will not be good.*

*Interviewer: As long as you can perform your daily activities at home, sir?*

*Patient: Yes, but I can no longer work, earn money, and provide for my family… (P12: 182-187)*

Observing these different goals of *ikhtiar* described by our participants reveals the variations in the understandings and practices of making an effort among Javanese Muslim communities.

### Being sincere (*ikhlas*/إِخْلَاص)

In our study, participants described two forms of being sincere (*ikhlas*). Based on their description, we define this value of *ikhlas* as a state or condition of being sincere, which entails an honest and genuine feeling. The first form of *ikhlas* was evident in the acceptance of the patient's condition, while the second form was the assumption of the caregiving role. Patients and their family caregivers, especially those who experienced the progression of the disease over a long period, demonstrated *ikhlas* as a sincere acceptance of the patient’s diagnosis and prognosis. The value of *ikhlas* allowed them, particularly the patient’s family, to be prepared to face the eventual death of their loved one.

*Patient’s son-in-law: If you see her condition, sometimes, it’s pitiful (melasi). If God decides to take her “home” (dipundut wangsul), we are already ikhlas.*

*Patient’s daughter: Yes, ikhlas, it (the disease) has been quite a long time.*

*Patient’s son-in-law: Her condition is hopeless; it has been like that for decades. It would be a miracle if she were cured.*

*(F07ab: 12-15)*

Healthcare workers observed that patients and families who have reached a state of *ikhlas* are easier to communicate and collaborate with.

*Interviewer: How did patients and families respond to your advice of not resuscitating the patient?*

*Gynecologic oncologist: It varies. But in most cases, because they are cancer patients, I usually inform them from the initial meeting that the condition can worsen suddenly, so regular check-ups are necessary. The journey can be long, so most of them have already accepted the condition. Except for distant relatives who never participated in medical consultations but suddenly appear and create tension. "My mother is not in a good condition; she is not being treated well!" In such situations, I can easily respond, "How long have you been with her? Why have you brought her when the condition is already (severe) like this?" I can counter that argument. It’s not that we don’t want to treat the patient, but the condition is already beyond treatment. (D08: 88-91)*

The second form of *ikhlas* was demonstrated when families performed the role of caregiving for their loved ones. This entails going the extra mile to care for them, such as taking them to various healthcare facilities and spending additional money to arrange for home care services. Family caregivers remained motivated to provide caregiving sincerely and wholeheartedly, even though they had to ‘sacrifice’ their time for other family members, including a spouse and children. With support from other family members, the main family caregiver could better perform his or her role of sincere caregiving.

*Interviewer: Does your husband understand and accept that you are busy taking care of your mother?*

*Patient’s daughter: Yes, he does. He sees my mother as not just his mother-in-law but also his own mother. He recognizes that the situation is as it is, with my mother being bedridden from a stroke. He told me, “It’s okay; I am ikhlas to take care of household chores. You should be ikhlas too, as you will be rewarded with good deeds for caring for our parents"; that is how he responds. (F01: 203-206)*

Both forms of *ikhlas* highlighted in our study emphasize the importance of sincerity, whether in accepting the patient’s fate (illness and prognosis) or caregiving. This state of being sincere was considered as a positive value by our participants.

### Being in a state of surrender (*pasrah*)

We learned that *pasrah* was used to describe a state of surrender to the conditions that participants were experiencing. We observed two different perceptions of *pasrah*. The first involves reliance on God and trusting God’s decisions, aligning with the Islamic notion of *tawakal.* This perception is used in a positive context to facilitate acceptance and coping. The second perception of *pasrah* is characterized by a sense of giving up and doing nothing, which was perceived negatively by our participants.

In the following interview with one healthcare worker, a psychologist demonstrated *pasrah* as praying to God and relying on the fate determined by God. She attempted to support patients and their families in adopting *pasrah,* which can be helpful for their psychological well-being.

*Interviewer: Many doctors are concerned about telling the truth regarding a patient’s incurable condition, is this concern related to the patient’s mindset focused on seeking a cure?*

*Psychologist: Well, most patients and their families have a curative mindset. They believe that seeing a doctor, getting a hemodialysis, or receiving chemotherapy will lead to a cure. We (the palliative team) explain to them that these treatments aim not only for cure, but also for achieving ‘healthiness’. This state of healthiness means they can still be empowered to continue their daily activities and maintain their mental well-being, avoiding psychological suffering. Instead of becoming frustrated with their painful condition, I advise them to pray and say, “Oh God, Ya Allah, I hope this pain will remove my previous sins.” By relying on God in this way, their perspective can be positively improved. (Psi01: 48-51)*

On the family side, *pasrah* is perceived as trusting God’s decision on whether the patient can be cured or not.

*Interviewer: The patient's condition is quite severe; as you said earlier, it is difficult to cure because it has been a while. What kind of hope do you have now?*

*Patient’s son-in-law: My only hope now is just pasrah and pasrah, that’s it. If God still grants her life, I hope she becomes healthier, at least. Because, you know, I feel pity for her seeing her like this. If God wants her to die, we are prepared and ikhlas as well. (F07: 11-12)*

Looking at her mother-in-law's condition, the participant emphasized it is up to God whether to extend her mother's life or not. He also mentioned that even if death is the result, he is ready to accept that fate. The participant used both *pasrah* and *ikhlas*, showing his acceptance of the patient's condition and his reliance on God's decision.

The second perception of *pasrah* involves giving up, not taking any action, and not attempting to do anything. Healthcare workers expressed concerns about being labeled as ‘*pasrah’* in this sense by patients or their families. This was particularly evident in situations where healthcare workers decided not to pursue aggressive treatment or chose not to resuscitate end-stage patients due to clinical and palliative considerations. Another situation that raised similar concern was discharging patients from the hospital when they had not yet visibly recovered, despite showing tangible improvement in their clinical conditions. The following quote illustrates one of these situations.

*Emergency room doctor: I have also faced complaints from the patient's family.*

*Interviewer: How did that happen? Were you perceived to be neglecting the patient?*

*Emergency room doctor: Well, yes, they thought I neglected the patient, but they also thought we did not do anything. We did not make any effort. They saw us as being pasrah. They wanted us to make a maximum effort. However, the idea of "maximum effort" in palliative care differs from their perspective. When I informed them about the severity of the condition, they filed a complaint. "The doctor said that my father will die. Is that true?!" It seems like there was some miscommunication, I think. (D02: 175-179)*

The following interview provides an example of how a patient’s tendency towards pessimistic behavior was perceived as *pasrah*, particularly by healthcare workers providing home care.

*Interviewer: Besides wound care, what else can we provide for patients at home in your opinion?*

*Homecare nurse: Actually, it depends on the patients themselves. If the patient is motivated and supported with good nutrition and routine medication, Inshaa Allah (By God’s will), the wound will improve faster. However, some patients are emotional. They are pasrah, as they don’t want to know the progression of the disease. Their pasrah attitude is more like, "If I get healed, so be it. If I die, so be it." This type of patient demotivates us to care for them. Their progress will usually be different compared to others who are more motivated.*

*Interviewer: Is it like a pessimistic behavior?*

*Home care nurse: Exactly. If they have a pessimistic behavior, it becomes challenging. No matter how much we try to motivate or inform them, they don’t respond. I have encountered this type of patient many times. (N04: 85-88)*

This quote shows how *pasrah* can also be perceived negatively as a pessimistic behavior. When patients exhibited pasrah behavior as described above, they appeared careless and lacking motivation to maintain their condition. It was challenging for health professionals to deal with patients or families who had a pessimistic outlook. It was perceived by the health professionals’ participant that the care recipients did not reciprocate the caregiving spirit. This phenomenon often left health professionals upset and saddened.

## Discussion

**Fig. 2:**
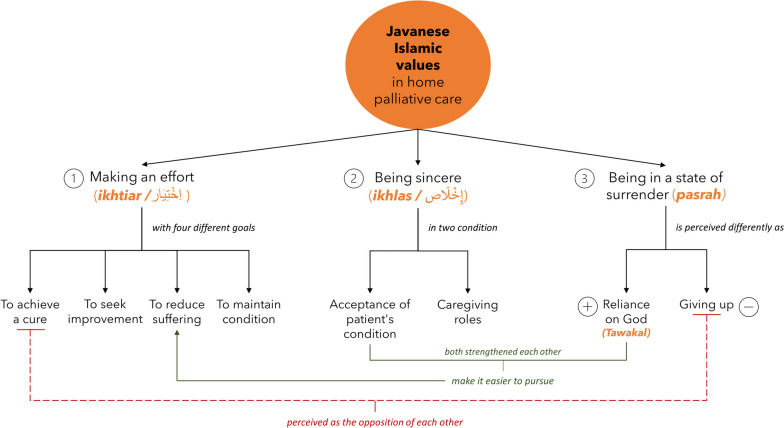
The relationship between categories and themes

We identified three distinct and specific values related to palliative care within a Javanese Muslim community in Indonesia. These values are interconnected and influence one another, sometimes providing mutual support. For instance, once patients and their families feel they have tried their best *(ikhtiar),* they can then sincerely (*ikhlas*) accept the patient's condition. Subsequently, they can rely on God (*tawakal*) for the outcome of their *ikhtiar*. On the other hand, the values may also conflict with each other. If patients and their families have not yet reached a state of *ikhlas*, they may continue to seek excessive or inappropriate medical care, which sometimes prevents them from accepting the reality. Health professionals said that the fear of being perceived as giving up by patients or their families put them in a dilemma of whether to pursue more aggressive treatment or not. The relation between these values in the study is illustrated in the conceptual map below (Fig. 2).

Our findings resonate with previous studies regarding Islamic values in palliative and end-of-life care trajectories. For example, a tension between fear, acceptance, and hope is commonly observed in patients and families with a Muslim background in Muslim and Non-Muslim majority countries [31–34]. Muslim health providers in different countries were also found to face difficulties in terminating treatment, rooted to their Islamic faith [35]. However, the specific values used in Banyumas, Indonesia provide a more specific understanding of how these values are interconnected. For instance, we argue that applying the notion of *ikhtiar* to different goals than attempts at cure. We revealed that *ikhtiar* was seen as a precondition to achieve *pasrah*, and how *ikhlas* helps to promote acceptance.

Previous studies conducted in Turkish and Moroccan communities residing in the Netherlands have indicated that these communities consider maximum curative treatment near the end of life as "good care", reflecting their preference for reaching a cure and doing everything possible [36]. Some Islamic families may ask for surgery even if it is clear that the patient will die, rather than waiting for the inevitable death [37]. Unfortunately, this hopeful waiting for a miracle often prolongs the suffering experienced by the patients [38]. The notion of striving and persistence was also reported in a previous study conducted within Muslim communities in the United Kingdom, where a commitment to steadfastness and hope was somehow driven by the belief that God’s power can provide a miraculous cure [32]. A previous study by Hendriks et al. highlighted the challenges faced by medical professionals when responding to the requests of patients with Islamic backgrounds, such as their wish to do "something” or engage in "all-out" medical care despite its risk and futility [37]. This phenomenon is aligned with our findings, where *ikhtiar,* especially aiming to achieve a cure, holds significant importance for patients and families. Health professionals should acknowledge this goal. However, when the nature of the disease precludes a cure, it may also be helpful to clarify that there are other equally important goals, such as reducing suffering and maintaining the patient's stable condition, which can improve the patient’s quality of life. Nevertheless, even with careful and sensitive communication from health professionals, resistance may still arise from patients and their families. The decision to forgo further medical treatment may be perceived as giving up, which is considered a less preferable, or even a forbidden action by Islamic believers, particularly when the doctrine of *ikhtiar* is strongly held [39].

One of the goals of *ikhtiar* mentioned by our participants is to reduce suffering. Even though suffering in Islam is considered a way to mend past sins and elevate one’s rank in the afterlife, Islam encourages believers to seek relief from suffering [40]. A study in Israel has explained the alignment between Islamic teachings and palliative care principles, particularly in the context of reducing pain and suffering. Islam teaches that God has explicitly instructed believers to seek treatment as every illness comes with its own remedies, except for death [39]. Islam emphasizes that death is not the enemy since it is an inevitable reality which everyone will eventually face. It is meant to be accepted as part of the divine plan, serving as a gateway to eternal life in the afterlife. Accepting death can also be seen as a component of *ikhlas*, as patients are encouraged to accept the inevitable end that awaits all human beings, regardless of when and how it may occur [39]. A previous study by Abu Khait and Lazenby [41] further supports the idea that accepting the end of life is facilitated by relying on God’s will, as embedded in the phrase “letting go, letting God”.

So, how do we use the concepts of *ikhtiar, ikhlas,* and *pasrah* to support palliative care and facilitate the integration of palliative care within Javanese Muslim communities? This is particularly needed when health professionals encounter situations where patients or their families wish to escalate treatment based on their Islamic values, even when such treatment offers no feasible benefits or may even cause more suffering. First, we suggest that it may be helpful to frame palliative care in alignment with the concept of *ikhtiar* by emphasizing that the pursuit of *ikhtiar* can also be carried out by applying palliative approach. This involves carefully managing symptoms of the disease and enhancing the patient’s quality of life. These various aspects of palliative care can be considered as part of *ikhtiar*. Recognizing the significance of achieving a cure, health professionals can continue curative treatment if it still outweighs the disadvantages. However, in cases where curative treatment only leads to further suffering and harm, health professionals can engage in discussions with patients and their families, sharing the ideas of the different goals of *ikhtiar* and introducing alternative ways of pursuing *ikhtiar*. By reaching a mutual understanding, patients, families, and health professionals can collaborate to alleviate suffering and improve quality of life, based on the best interests of the patient.

Second, we want to suggest how the concept of *ikhlas* can be applied to help patients and families accept unfavorable diagnoses and prognoses. At times, they may persist in hoping for a miracle and struggle to embrace the realities of their situation. To address this, we suggest that health professionals first acknowledge this concern and then assist them in cultivating *ikhlas* and relying on God's decision. Encouraging patients and families to embrace *ikhlas* can help them to sincerely accept the fate determined by God as the ultimate provider, who possesses divine wisdom. This understanding can help alleviate anxiety and foster a sense of contentment, particularly when facing a poor prognosis. It becomes easier for patients and their families to embrace the realities and embark on another *ikhtiar* to strive for a better quality of life and alleviate suffering, all while placing their faith in God's decision. Providing some form of spiritual support can be beneficial in helping patients and families find meaning in life and its ending, especially after enduring difficult and challenging situations. Additionally, health professionals can assist Javanese Muslim family caregivers to cultivate *ikhlas* in their caregiving roles. This enables them to provide sincere care that prioritizes the patient’s well-being without being motivated by external worldly recognition. However, it is crucial for health professionals to be attuned to the subtle messages conveyed by patients or families when they mention *ikhlas*, as it may mask underlying burdens and unspoken needs. In-depth discussions about religious and spiritual needs should occur in a relaxed setting, such as the patient's home during a home care visit, to foster a deeper connection with patients and families and create a safe space where these needs can be openly addressed.

Third, we propose to elaborate further on the value of *pasrah* and its position within palliative care. When patients and families perceive palliative care as giving up and not taking any action, health professionals can try the strategy of clarifying that palliative care does not mean giving up, but rather aligns with either the concept of *ikhtiar* or *ikhlas*. On the other hand, when patients or families showed their preference to not pursue further medical treatment, this can also be perceived as the pessimistic *pasrah* by healthcare professionals who were not aware of palliative care concepts. This mismatch between patient’s preference and the delivered care could come from the currently low level of palliative care knowledge among Indonesian health professionals [42].

Due to the coexistence of two different perceptions of *pasrah* in Javanese Muslim communities – giving up and relying on God – health professionals need to be cautious when using the term *pasrah* when communicating with patients and their families. One alternative, particularly for Javanese Muslim communities, could be introducing the word *tawakal* as it carries less ambiguity. *Tawakal* encompasses both the significance of making an effort (*ikhtiar*) as a prerequisite of relying on God’s will, thereby negating the notions of passivity and negligence.

We reiterate the prophetic teaching of “*Tie the camel first, then rely on God*” in this context. The teaching of Prophet Muhammad, where he encouraged Muslims to “tie their camel”, represents the concept of *ikhtiar*, but this time, as an active effort to improve quality of life and alleviate patient’s suffering. The subsequent step of “relying on God” after tying the camel represents patients and families placing trust in God’s plan after trying their best (*ikhtiar*). Thus, both values can complement each other and contribute to the integration of palliative care within Javanese Muslim communities in Indonesia.

## Strengths and limitations

This paper offers an empirical understanding of how values influence the care practices of patients, families, and health professionals in a palliative setting. Through a detailed and meticulous description of this ethnographic study, the influence of Javanese-Islamic values is evident in the efforts made by the participants, the sincerity of their actions, and their overall perception of palliative care. Our study provides further strategies to align palliative care concepts with Javanese-Islamic values that maybe helpful in the effective communication of introducing palliative care.

Through specification, detailed, and nuanced description of the research, we provided tools to help the reader consider whether our findings can be compared and applied in their specific setting [43]. Even though the reader could assume that all Muslim patients employ these values, the application of Islamic values in palliative and end-of-life care varies among Muslim patients due to cultural, social, and scholarly influences [44]. Further research is needed to compare how Islamic communities in different countries engage with these concepts.

## Conclusions

We argue that incorporating local values, in this case Javanese Islamic values, into care conversations is helpful for patients and families in this context, as it allows for the alignment of palliative care concepts with local values. To facilitate this alignment, we propose three conceptual strategies: integrating quality of life into the goals of ikhtiar; cultivating ikhlas; and modifying the negative perception of pasrah as giving up. People from different religious and cultural backgrounds in Indonesia might also learn from these ideas, as these terminologies have been adapted into Bahasa Indonesia and widely used across Indonesian communities. These strategies can assist health professionals in making palliative care understandable and acceptable to patients and families, enabling care that prioritizes the quality of life rather than futile attempts at a cure.

## Data Availability

The datasets used and/or analyzed during the current study are not publicly available due to the ethical and privacy reasons around the sensitive nature of the material but are available from the corresponding author (RBW) on reasonable request.
